# Assessing long-term survival and hospitalization following transvenous lead extraction in patients with cardiac resynchronization therapy devices: A propensity score–matched analysis

**DOI:** 10.1016/j.hroo.2021.10.006

**Published:** 2021-10-30

**Authors:** Vishal S. Mehta, Hugh O’Brien, Mark K. Elliott, Baldeep S. Sidhu, Justin Gould, Anoop K. Shetty, Steven Niederer, Christopher A. Rinaldi

**Affiliations:** ∗ Cardiology Department, Guy’s and St Thomas’ NHS Foundation Trust, London, United Kingdom; † School of Biomedical Engineering and Imaging Sciences, King’s College London, London, United Kingdom

**Keywords:** Cardiac resynchronization therapy, CRT, Hospitalization, Mortality, Propensity score matching, Transvenous lead extraction

## Abstract

**Background:**

Longer-term outcomes of patients post transvenous lead extraction (TLE) are poorly understood in patients with cardiac resynchronization therapy (CRT) devices.

**Objectives:**

A propensity score (PS)–matched analysis evaluating outcomes post TLE in CRT and non-CRT populations was performed.

**Methods:**

Data from consecutive patients undergoing TLE between 2000 and 2019 were prospectively collected. Patients surviving to discharge and reimplanted with the same device were included. The cohort was split depending on presence of CRT device. Associations with all-cause mortality and hospitalization were assessed by Kaplan-Meier estimates. An exploratory endpoint was evaluated whether early (<7 days) or late (>7 days) reimplantation was associated with poorer outcomes.

**Results:**

Of 1005 patients included, 285 (25%) had a CRT device. Median follow-up was 57.00 [27.00–93.00] months, age at explant was 67.7 ± 12.1 years, 83.3% were male, and 54.4% had an infective indication for TLE. PS was calculated using 43 baseline characteristics. After matching, 192 CRT patients were compared with 192 non-CRT patients. In the matched cohort, no significant difference with respect to mortality (hazard ratio [HR] = 1.01, 95% confidence interval [CI] [0.74–1.39], *P* = .093) or hospitalization risk (HR = 1.2, 95% CI [0.87–1.66], *P* = .265) was observed. In the matched CRT group, late reimplantation was associated with increased mortality (HR = 1.64, [1.04–2.57], *P* = .032) and hospitalization risk (HR = 1.57, 95% CI [1.00–2.46], *P* = .049].

**Conclusion:**

Outcomes of CRT patients post TLE are similarly as poor as those of non-CRT patients in matched populations. Reimplantation within 7 days was associated with better outcomes in a CRT population but was not observed in a non-CRT population, suggesting prolonged periods without biventricular pacing should be avoided.

**Key Findings**

▪ This is the largest matched analysis of mortality and clinical outcomes of patients with and without cardiac resynchronization therapy (CRT) devices following transvenous lead extraction (TLE).
▪ In an unmatched analysis, patients with CRT devices post TLE were more likely to die and be readmitted to hospital for any cardiovascular cause.
▪ In a matched analysis, patients with and without CRT devices post TLE had similar outcomes with respect to mortality and hospitalization.
▪ Delayed reimplantation following TLE in the CRT group was associated with greater risk of mortality and hospitalization. This was not observed in the non-CRT group. This suggests minimizing time without biventricular pacing following TLE in a CRT population is desirable.

## Introduction

The rise in the use of intracardiac implantable electronic devices (CIEDs) has been paralleled by an increase in the number of procedures required for the removal of such devices and their associated leads.^1^ Transvenous lead extraction (TLE) forms the basis of the management of infected CIEDs and malfunctioning and redundant leads.^2^ High procedural success rates with low rates of major in-hospital complications, as achieved in the European Lead Extraction ConTRolled Registry (ELECTRa), demonstrate a complete clinical success at 96.7% and an in-hospital major complication rate at 1.7%.^3^ Overall hospital mortality was low at 1.4% with a procedural-related mortality of 0.5%. The outcomes for the subgroup of patients who have TLE procedures with cardiac resynchronization therapy (CRT) devices is less well understood. CRT is an effective therapy to improve symptoms and reduce mortality in patients with dyssynchronous heart failure; however, these patients have a higher morbidity and mortality rate related to poorer left ventricular ejection fraction (LVEF) and comorbidity burden. Similarly, the number of CRT devices implanted with left ventricular (LV) leads has been paralleled by an increased requirement for CRT system extraction.^4^ Current evidence suggests that there is no significant difference in acute complications or 30-day mortality associated with CRT system extraction.^5^ Less is understood regarding long-term outcomes regarding mortality and morbidity following TLE in this group. In addition, the impact of delayed reimplantation of a CRT device following TLE is poorly understood, despite the theoretical risk of negative reverse remodeling^6^ or acute hemodynamic compromise^7^ caused by the absence of biventricular (BiV) pacing. We hypothesized that patients had poorer outcomes who had a CRT device vs non-CRT device; however, it was unclear if matching the baseline characteristics would maintain this effect. In addition, we hypothesized that delayed reimplantation post TLE in a CRT population would result in poorer outcomes compared to non-CRT populations. We studied data from a single, high-volume tertiary referral center for TLE, regarding long-term outcomes in a CRT and non-CRT population.

## Methods

### Data collection

All consecutive patients undergoing TLE in a high-volume center in the UK were prospectively recorded onto a computer database between October 2000 and November 2019. Multiple parameters were recorded, including demographics, extraction indication, device and lead type, comorbidities, biochemistry and pathology results, procedural success, major complications, and technical extraction information. Patients reimplanted with the same device and surviving to discharge following TLE were included. Only the most recent entry for patients with multiple TLEs during the study period were included. Mortality was recorded retrospectively by linking unique patient registration numbers (National Health Service [NHS] numbers) and the Office for National Statistics mortality data updated as of February 2020.^8^ Hospital readmission information was obtained from the source data feeding directly to the Hospital Episodes Statistics national database, which records all NHS hospital-based activity in England and has been validated as an accurate way of recording medical activity and is used for allocating resources based on needs in the NHS.^9^ Any cardiovascular cause of inpatient admission was identified as the primary outcome measure of hospitalization, as defined by the World Health Organization International Classification of Diseases (ICD-10-CM) coding system (ICD-10-CM codes: Diseases of the circulatory system: ICD I00-199; Heart failure: I50; Complications of cardiac and vascular prosthetic devices: ICD T82).^10^ The database collection and analysis were approved by the Institutional Review Board of Guy’s and St Thomas’ Hospital.

### Definitions

TLE was defined as per the EHRA and HRS guidelines.^11^ The 2018 EHRA guidelines defined the extraction indication, procedural success, and complication rate.^2^ The extraction procedure undertaken at this center has been described in detail elsewhere.^12^ If there was more than 1 indication for lead extraction or original implantation indication, this was counted independently. Number of previous device interventions was defined as the number of CIED procedures undertaken on the patient prior to the recorded lead extraction. Lead dwell time was calculated as the oldest targeted lead in situ at time of extraction. Follow-up time and age were calculated from date of TLE. Major cardiovascular comorbidities were recorded. Glomerular filtration rate was estimated (eGFR) by the MDRD 4-variable equation.^13^

### Statistical analysis

Missing data for variables of interest were handled by multiple imputation with chained equations and the multiple imputed data frames were merged into a single data frame by computing the mean or selecting the most likely imputed value (R-packages mice and sjmisc; 10 imputed datasets).^14^ The propensity score (PS) for the CRT group was calculated by a logistic regression model using 43 clinically relevant covariates. CRT patients were matched 1:1 to non-CRT patients by their PSs, using the nearest-neighbor method with a caliper of 0.10 and no replacements. Variables either included in the multiple imputation models or considered for PS calculation are shown in Table 1. The ability of the matching to balance baseline characteristics in the CRT vs non-CRT group was assessed by absolute standard differences, with a value of <10% considered as not significant.^15^

**Table 1:** Baseline characteristics of the unmatched and the propensity score–matched cohort

Variable	Unmatched cohort				Matched Cohort			
	Non-CRT group (n = 720)	CRT group (n = 285)	*P* value	% Missing	Non-CRT group (n = 192)	CRT group (n = 192)	*P* value	SD
Follow-up time in months, median [IQR]†	61.50 [31.75, 99.25]	38.00 [21.00, 73.00]	<.001	0.0%	47.00 [27.75, 76.75]	43.50 [24.00, 75.50]	.469	-
Male, n (%)†‡	489 (67.9)	242 (84.9)	<.001	0.0%	160 (83.3)	160 (83.3)	1	0.06
Age at explant, mean (SD)†‡	63.98 (15.92)	67.96 (10.70)	<.001	0.0%	67.81 (12.92)	67.62 (11.18)	.876	1.25
Time to reimplant, days†‡	0.00 [0.00, 10.00]	7.00 [0.00, 12.00]	.029	0.8%	6.00 [0.00, 11.00]	6.00 [0.00, 11.00]	.897	0.44
Reimplanted on same day, n (%)†‡	365 (50.7)	128 (44.9)	.113	0.8%	86 (44.8)	87 (45.3)	1	0.87
Reimplanted within 7 days, n (%)†‡	470 (65.3)	159 (55.8)	.006	0.8%	118 (61.5)	114 (59.4)	.754	0.93
Lead dwell time (months), median [IQR]†‡	70.30 [21.73, 125.78]	56.30 [21.00, 97.00]	.009	2.8%	65.95 [27.30, 121.22]	57.40 [21.62, 107.93]	.205	1.11
Lead dwell time (years), median [IQR]†‡	5.90 [1.80, 10.50]	4.70 [1.80, 8.10]	.01	4.5%	5.50 [2.27, 10.10]	4.80 [1.80, 9.00]	.206	1.11
CRTP, n (%)	-	61 (21.4)	-	0.3%	-	53 (27.6)	-	-
CRTD, n (%)	-	224 (78.6)	-	0.3%	-	139 (72.4)	-	-
No. of patients without LV lead extracted (as % of CRT devices)	-	33 (11.6)	-	0.0%	-	15 (7.8)	-	-
ICD, n (%)	286 (39.7)	-	-	0.0%	114 (59.4)	-	<0.001	-
Pacing device, n (%)	434 (60.3)	-	-	0.0%	78 (40.6)	0 (0.0)	<0.001	-
Local infection, n (%)†‡	255 (35.4)	108 (37.9)	.506	0.1%	81 (42.2)	75 (39.1)	.603	0.90
Systemic infection, n(%)†‡	114 (15.8)	45 (15.8)	1	0.0%	25 (13.0)	28 (14.6)	.767	0.70
Any infection, n (%)†‡	369 (51.2)	153 (53.7)	.531	0.0%	106 (55.2)	103 (53.6)	.838	0.97
Lead dysfunction, n (%)†	233 (32.4)	103 (36.1)	.284	0.0%	46 (24.0)	70 (36.5)	.011	-
Functional lead, n (%)†	21 (2.9)	3 (1.1)	.13	0.3%	6 (3.1)	3 (1.6)	.5	-
Lead complication, n (%)†	52 (7.2)	21 (7.4)	1	0.0%	20 (10.4)	12 (6.2)	.196	-
Lead access, n (%)†	39 (5.4)	9 (3.2)	.177	0.5%	22 (11.5)	8 (4.2)	.013	-
Lead pain, n (%)†	12 (1.7)	2 (0.7)	.38	0.3%	2 (1.0)	2 (1.0)	1	-
Other indication, n (%)†	72 (10.0)	21 (7.4)	.239	0.0%	23 (12.0)	11 (5.7)	.048	-
LVEF, mean (SD)†‡	47.43 (12.09)	35.48 (12.42)	<.001	12.3%	38.09 (13.37)	37.70 (12.42)	.764	1.07
Ischemic heart disease, n (%)†‡	225 (31.2)	167 (58.6)	<.001	2.7%	100 (52.1)	107 (55.7)	.539	0.98
CABG, n (%)†‡	70 (9.7)	64 (22.5)	<.001	2.9%	37 (19.3)	37 (19.3)	1	0.32
Valve disease, n (%)†‡	67 (9.3)	31 (10.9)	.523	3.0%	25 (13.0)	23 (12.0)	.877	0.64
Heart failure, n (%)†‡	152 (21.1)	232 (81.4)	<.001	2.6%	141 (73.4)	140 (72.9)	1	0.17
Diabetes mellitus, n (%)†‡	80 (11.1)	76 (26.7)	<.001	3.6%	38 (19.8)	42 (21.9)	.706	0.66
Hypertension, n (%)†‡	248 (34.4)	139 (48.8)	<.001	3.7%	87 (45.3)	88 (45.8)	1	1.11
Peripheral vascular disease, n (%)†‡	16 (2.2)	22 (7.7)	<.001	3.6%	11 (5.7)	8 (4.2)	.638	0.29
Stroke, n (%)†‡	45 (6.2)	33 (11.6)	.007	3.4%	17 (8.9)	19 (9.9)	.861	0.59
Chronic respiratory disease, n (%)†‡	69 (9.6)	55 (19.3)	<.001	3.6%	27 (14.1)	29 (15.1)	.885	0.63
Chronic kidney disease, n (%)†‡	98 (13.6)	88 (30.9)	<.001	2.3%	49 (25.5)	53 (27.6)	.729	0.70
Total number of comorbidities, n (%)†‡			<0.001	0.0%			0.549	0.93
0	258 (35.8)	10 (3.5)			11 (5.7)	10 (5.2)		
1	152 (21.1)	35 (12.3)			35 (18.2)	31 (16.1)		
2	138 (19.2)	61 (21.4)			43 (22.4)	49 (25.5)		
3	91 (12.6)	61 (21.4)			43 (22.4)	38 (19.8)		
4	52 (7.2)	55 (19.3)			34 (17.7)	31 (16.1)		
5	16 (2.2)	35 (12.3)			13 (6.8)	19 (9.9)		
6	10 (1.4)	24 (8.4)			10 (5.2)	14 (7.3)		
7	3 (0.4)	4 (1.4)			3 (1.6)	0 (0.0)		
Creatinine level (mg/dL), mean [SD]†‡	89.00 [75.00, 110.00]	108.00 [86.00, 136.00]	<.001	1.6%	100.00 [83.00, 127.25]	102.00 [83.00, 129.25]	.892	0.90
eGFR (mL/min/1.73 m^2^), mean (SD)†‡	70.45 (18.61)	60.34 (19.90)	<.001	1.6%	63.88 (20.15)	63.11 (19.98)	.707	1.09
eGFR <60 mL/min/1.73 m^2^, n (%)†‡	194 (26.9)	135 (47.4)	<.001	1.6%	73 (38.0)	80 (41.7)	.532	0.97
History of previous extraction, n (%)†‡	72 (10.0)	46 (16.1)	.009	0.0%	25 (13.0)	27 (14.1)	.881	0.57
No. of previous device interventions, n (%)†‡			.038	0.1%			.506	0.56
0	647 (89.9)	239 (83.9)			166 (86.5)	165 (85.9)		
1	44 (6.1)	25 (8.8)			15 (7.8)	12 (6.2)		
2	28 (3.9)	21 (7.4)			10 (5.2)	15 (7.8)		
3 or more	1 (0.1)	0 (0.0)			1 (0.5)	0 (0.0)		
Superior approach, n (%)†	703 (97.6)	282 (98.9)	.276	0.0%	189 (98.4)	189 (98.4)	1	-
Manual traction only, n (%)†‡	141 (19.6)	53 (18.6)	.788	0.0%	30 (15.6)	36 (18.8)	.499	0.72
Nonpowered tool only, n (%)†‡	121 (16.8)	58 (20.4)	.218	0.0%	35 (18.2)	34 (17.7)	1	0.76
Powered tools only, n (%)†‡	81 (11.2)	30 (10.5)	.827	0.0%	16 (8.3)	19 (9.9)	.723	0.56
Inferior approach, n (%)†‡	68 (9.4)	36 (12.6)	.167	0.3%	23 (12.0)	26 (13.5)	.76	0.74
Complete removal, n (%)†‡	635 (88.2)	242 (84.9)	.193	0.0%	172 (89.6)	164 (85.4)	.28	0.64
Partial removal, n (%)†‡	64 (8.9)	35 (12.3)	.131	0.0%	15 (7.8)	21 (10.9)	.381	0.54
Clinical failure, n (%)†‡	6 (0.8)	3 (1.1)	1	0.0%	1 (0.5)	2 (1.0)	1	0.15
Minor complications, n (%)†‡	59 (8.2)	26 (9.1)	.726	0.0%	15 (7.8)	15 (7.8)	1	0.15
Major complications, n (%)†‡	16 (2.2)	2 (0.7)	.169	0.0%	2 (1.0)	2 (1.0)	1	0.02

Continuous variables are presented as mean (standard deviation) or median [interquartile range] as appropriate, categorical as frequency (%). The *t* test was used to compare CRT subjects vs non-CRT subjects for continuous variables; the Fisher exact test was used for categorical variables. Standardized differences (SD) are defined as the difference in means, proportions, or ranks divided by the mutual standard deviation.

CABG = coronary artery bypass graft; CRTD = cardiac resynchronization therapy defibrillator; CRTP = cardiac resynchronization therapy pacemaker; eGFR = estimated glomerular filtration rate; ICD = implantable cardiac defibrillator; LV = left ventricular; LVEF = left ventricular ejection fraction.

† Variables were included in the multiple imputation models together with the outcomes all-cause death and hospitalization.

‡ Variables were used for the calculation of propensity scores.

Baseline variables of the matched cohort were compared by calculating standardized mean differences and the χ^2^ test, Student *t* test, or Mann-Whitney *U* test when appropriate. Primary outcomes in this analysis were overall survival and time to first cardiovascular hospitalization at follow-up. Kaplan-Meier method was used to estimate survivor functions in the CRT vs non-CRT group, with a secondary outcome analysis dependent on whether patients were reimplanted within or after 7 days of initial TLE. A subanalysis of the matched CRT and non-CRT groups was undertaken with the same outcomes assessed as above. Univariable Cox (proportional hazard) regression was performed, and the results are presented as hazard ratio (HR) [95% confidence interval (CI)], *P* value.

## Results

### Study cohort

Between October 2000 and November 2019, 1171 consecutive patients underwent TLE at the reference center. After the inclusion criteria were applied, 1005 patients were eligible. Of these, 285 (28.4%) patients had a CRT device. After PS matching, the analysis was restricted to 384 patients, 192 in both the CRT and non-CRT groups.

### Baseline characteristics

Baseline characteristics are summarized in Table 1. In the overall cohort, mean age was 65.1 ± 14.7 years, 72.7% were male, and 51.9% had a TLE for an infective indication. Median [interquartile range] lead dwell time was 5.40 [1.80–9.80] years, 28.5% had an ICD, 43.2% had a permanent pacemaker, and the remainder had a CRT-D/P device at time of TLE. Most of the baseline characteristics were differently distributed in the CRT vs non-CRT group. CRT patients were older (68 ± 10.7 vs 64 ± 15.6 years, *P* < .001), had a higher mean number of comorbidities (3.18 vs 1.49, *P* < .001), had poorer renal function (108.00 [86.00–136.00] vs 89.00 [75.00–110.00] mg/dL, *P* < .001), and had lower LVEF (35.5 ± 12.4 vs 47.4 ± 12.1, *P* < .001). The CRT group had a shorter lead dwell time (4.70 [1.80–8.10] vs 5.90 [1.80–10.50] years, *P* = .01) and were less likely to have their device reimplanted within 7 days of TLE procedure (n = 159, 55.8% vs n = 470, 65.3%, *P* = .006). The CRT group also had a longer time to reimplantation (*P* = .029), and were more likely to have had a previous device intervention (*P* = .038). After PS matching, baseline characteristics considered for PS calculation were equally distributed between the 2 study groups, with well-matched PS in both groups (Supplemental Figure 1).

### Outcome analysis

#### All-cause mortality

**Figure 1:**
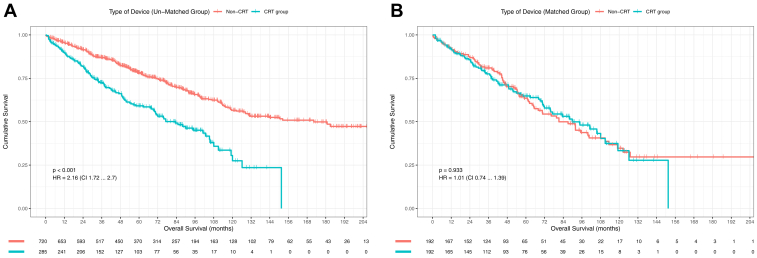
Kaplan-Meier survival probability for mortality in patients depending on type of device explanted. **A:** Unmatched cohort. **B:** Matched group. CRT = cardiac resynchronization therapy.

Figure 1 illustrates survival probability for all-cause mortality.

In the overall cohort, during long-term follow-up with a median of 57.00 [27.00–93.00] months, 345 (34.3%) patients died. Kaplan-Meier survival analysis demonstrated a survival probability of 93.4% at 1 year, 88.4% at 2 years, 73.1% at 5 years, and 50.4% at 10 years. At follow-up a higher proportion of patients died in the CRT vs non-CRT group (43.9% vs 30.6%, *P* < .001), with survival probability of 88.9% vs 97.1% at 1 year, 80.7% vs 91.4% at 2 years, 59.3% vs 78.3% at 5 years, and 27.6% vs 56.7% at 10 years. Overall unadjusted hazard ratio (HRs) for mortality and 95% CIs in the CRT group were HR = 2.16, 95% CI [1.72–2.70], *P* < .001].

In the matched cohort, during long-term follow-up with a median of 46.00 [25.00–76.25] months, 159 (41.4%) patients died. At follow-up a similar proportion of patients died in the matched CRT vs non-CRT group (40.1% vs 42.7%, *P* = .68) with survival probability of 91.4% vs 91.5% at 1 year, 83.9% vs 86.9% at 2 years, 65.0% vs 63.6% at 5 years, and 33.5% vs 34.9% at 10 years. Similar unadjusted HR were observed for the matched CRT group (HR = 1.02, 95% CI [0.74–1.39], *P* = .933).

#### Cardiovascular hospitalization

**Figure 2:**
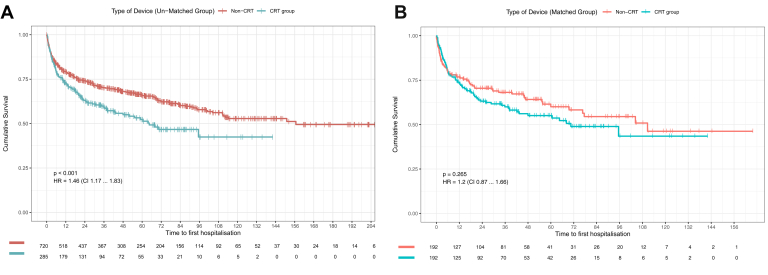
Kaplan-Meier survival probability for hospitalization in patients depending on type of device explanted. **A:** Unmatched cohort. **B:** Matched group. CRT = cardiac resynchronization therapy.

Figure 2 illustrates survival probability for cardiovascular hospitalization.

In the overall cohort during long-term follow-up, 371 (36.9%) patients were hospitalized. Kaplan-Meier survival analysis demonstrated a freedom from hospitalization probability of 76.7% at 1 year, 71.0% at 2 years, 62.2% at 5 years, and 50.1% at 10 years. At follow-up a higher proportion of patients were hospitalized in the CRT vs non-CRT group (58.9% vs 44.9%, *P* < .001), with survival probability of 71.6% vs 78.7% at 1 year, 62.8% vs 74.0% at 2 years, 51.6% vs 65.9% at 5 years, and 42.8% vs 53.1% at 10 years. Overall unadjusted HR and 95% CIs for hospitalization in the CRT group were greater than in the non-CRT group: HR = 1.46, 95% CI [1.17–1.83], *P* < .001.

In the matched cohort during long-term follow-up, 147 (38.3%) patients died. At follow-up a similar proportion of patients were hospitalized in the matched CRT vs non-CRT group (41.1% vs 35.4%, *P* = .294), with hospitalization probability of 72.2% vs 76.3% at 1 year, 63.3% vs 70.6% at 2 years, 54.0% vs 60.4% at 5 years, and 43.7% vs 46.5% at 10 years. Similar unadjusted HR were observed for the matched CRT group for risk of hospitalization (HR = 1.20, 95% CI [0.87–1.66], *P* = .265].

### Subgroup analysis

#### Reimplantation timing

In the subgroup analysis within the matched cohorts, an analysis of survival probability with respect to mortality and hospitalization following TLE was performed. There were similar baseline characteristics between the late reimplantation groups in the matched CRT and non-CRT groups, with similar infective indications for TLE (local: 64.1% vs 64.9%; systemic: 26.9% vs 27.0%; any infection: 91.0 vs 91.9%), eGFR (61.6 vs 61.9 mL/min/1.73 m^2^), LVEF (38.4% vs 40.1%), and age at explant (69.2 vs 70.0 years) (Supplemental Table 1).

**Figure 3:**
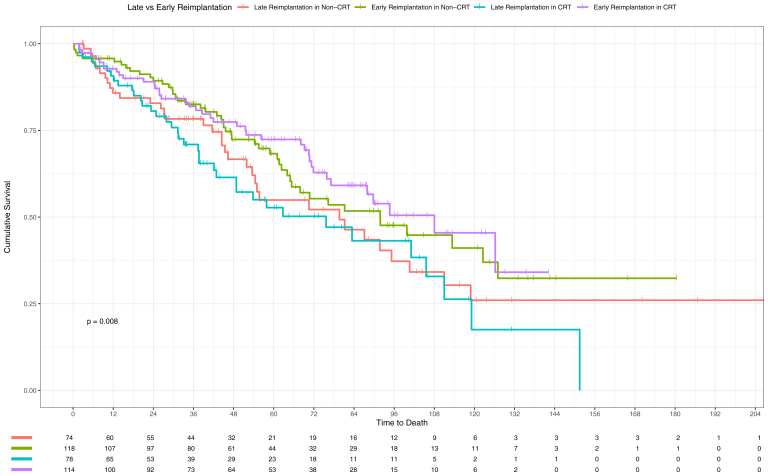
Kaplan-Meier survival probability for mortality in patients depending on timing for reimplantation post transvenous lead extraction in subgroup analysis of matched groups. CRT = cardiac resynchronization therapy.

**Figure 4:**
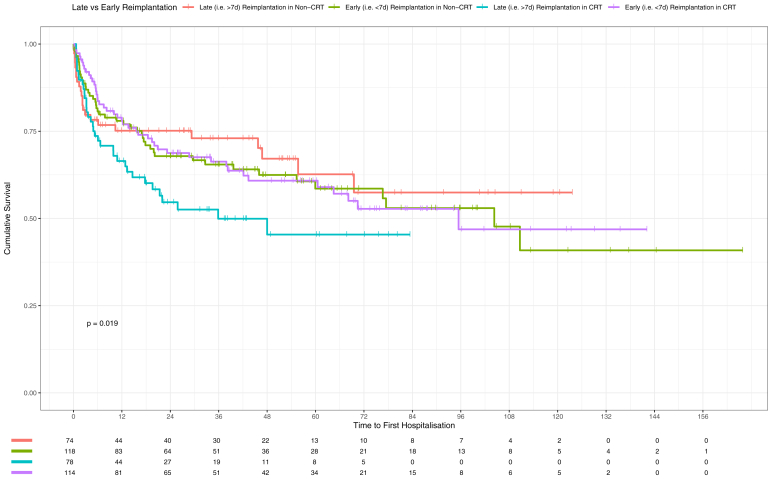
Kaplan-Meier survival probability for hospitalization in patients depending on timing for reimplantation post transvenous lead extraction in subgroup analysis of matched group. CRT = cardiac resynchronization therapy.

Within the matched non-CRT group, there was no significant difference with regard to risk if reimplantation occurred late (ie, 7 days after TLE procedure), with an unadjusted HR for death of HR = 1.33, 95% CI [0.86–2.05], *P* = .208, and for hospitalization HR = 1.14, 95% CI [0.69–1.89], *P* = .601. Within the matched CRT group, there was a significant difference with regard to risk associated with late reimplantation, with an unadjusted HR for death of HR = 1.64, 95% CI [1.04–2.57], *P* = .032 and for hospitalization HR = 1.57, 95% CI [1.00–2.46], *P* = .049. There was no evidence of differences in risk of mortality (*P* = .576) or hospitalization (*P* = .911) between the early reimplantation groups in the CRT and non-CRT groups. There was increased risk of hospitalization in the late reimplantation group in the CRT group vs non-CRT group (HR = 1.71, 95% CI [1.01–2.9], *P* = .048) (Figures 3 and 4).

#### Risk depending on cause of hospitalization

**Figure 5:**
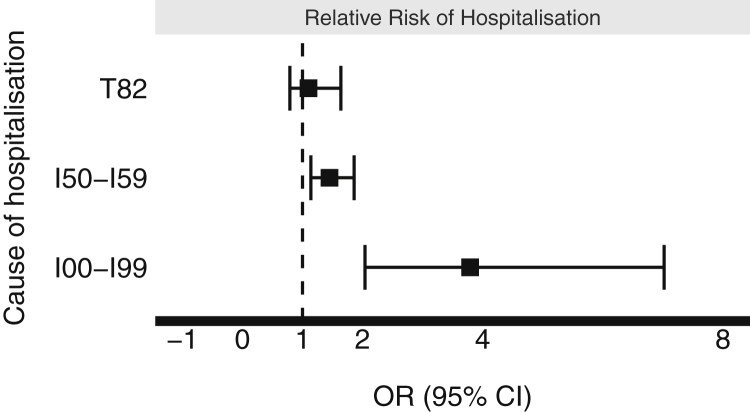
Cause-of-hospitalization analysis. Forest plot assessing relative risk of hospitalization for a specified cause following transvenous lead extraction in patients with cardiac resynchronization therapy (CRT) devices compared to non-CRT devices in the unmatched cohorts.

There was a greater risk of hospitalization associated with TLE in the CRT group compared to the non-CRT group with regard to any cardiovascular cause (ICD-10 I00–I99 codes) for hospitalization (relative risk [RR] 3.79, 95% CI [2.04–7.02], *P* < .001) or heart failure decompensation (ICD I50–I59 codes) (RR 1.45, 95% CI [1.14–1.86], *P* = .004). No significant difference was identified with respect to risk of device-related complications requiring hospitalization (RR 1.13, 95% CI [0.79–1.64], *P* = .515) (Figure 5).

## Discussion

An understanding of mortality and morbidity at follow-up post TLE in the CRT population is important to evaluate the longer-term implications of the procedure. To our knowledge, this analysis is the largest registry analysis to date evaluating mortality and morbidity outcomes following TLE in patients who survive to discharge and are reimplanted with the same device.

The main findings are that:

(1) The baseline characteristics of patients undergoing TLE in the CRT group are significantly different from the non-CRT group, and this is reflected in a higher risk of mortality and cardiovascular hospitalization following TLE.
(2) In a matched cohort, CRT and non-CRT patients had similar outcomes with respect to mortality and hospitalization risk post TLE.
(3) Following TLE, CRT patients had a higher risk of hospitalization for any cardiovascular cause or heart failure; however, there was no increased risk of hospitalization owing to a device-related complication.
(4) Reimplantation within 7 days was associated with better outcomes in a matched population in patients with a CRT device compared to a non-CRT population.

Few studies have compared long-term outcomes of patients following TLE specifically evaluating patients with CRT and non-CRT devices. Larger registry analyses have not evaluated outcomes beyond early complications and mortality in both CRT and non-CRT cohorts, including the ELECTRa study^3^ and the Cleveland Clinic series of 5000 TLEs.^16^ Data from the same reference center by Gould and colleagues,^5^ utilizing a smaller cohort of patients, has demonstrated no significant difference in 30-day mortality rates between CRT (3.0%, n = 7) and non-CRT patients (2.0%, n = 14) (*P* = .443). This study also evaluated outcomes using case-control matching, which also demonstrated no significant difference in 30-day outcomes; however, only 185 patients were included in each group, and they were matched only for 4 variables (lead dwell time, age, renal impairment, and systemic infection), whereas the current analysis matched for 43 variables (Table 1). Zucchelli and colleagues^17^ demonstrated a 1-year mortality of 5.5% in a CRT population post TLE, whereas our study demonstrated higher incidence of mortality of 11.1%. In a more recent study, Nishii and colleagues^7^ compared the prognosis of patients who had severe LV systolic dysfunction (SLVD) compared to those who did not. While not looking specifically at patients with CRT devices, they demonstrated that those with SLVD were not more likely to die at 30 days (97.2% vs 99.4%, *P* = .215) or 1 year (80.6% vs 91.5%, *P* = .053) post TLE.^7^ They also identified that patients with SLVD were more likely to require additional hemodynamic support, such as temporary CRT therapy pacing (27.8% vs 1.2%; *P* < .001), which may attest to the findings in our study identifying poorer outcomes for those who had delayed reimplantation. Of note, this study only included 36 patients with SVLD, out of a total cohort of 200 patients, whereas our study utilizes data from 1005 patients. Few studies have evaluated cardiovascular hospitalization as an endpoint in CRT patients post TLE. Regoli and colleagues^18^ identified 37.0% requiring hospitalization and 23.9% dying at a median follow-up of 21 months post TLE, which compared similarly to our study at the same follow-up time (hospitalization: 34.9%; mortality: 16.5%).

Most published data involving PS matching in patients with CRT has been to compare outcomes of CRT cohorts with and without defibrillator devices,^19^^,^^20^ with only 1 study utilizing PS matching in patients following TLE.^21^ This study is the first to match CRT and non-CRT patients post TLE. Matching resulted in an increase in mean age at explant (64.0 to 67.8 years), total number of comorbidities (1.49 to 2.78 comorbidities), and reduction in LVEF (47.4% to 37.7%) and eGFR (70.5 to 63.9 mL/min/1.73 m^2^) of the non-CRT group. In the unmatched cohort, CRT patients were at significantly increased risk of any cardiovascular hospitalization and mortality, with an increased relative risk of heart failure hospitalization, compared to a non-CRT population. Matching resulted in similarly poor outcomes in the CRT and non-CRT group, which suggests that all patients with a greater comorbidity burden, regardless of whether they have a CRT, may benefit from closer evaluation following TLE. This could confer significant cost savings for healthcare services, which can tailor services to reduce risk of hospitalization in these at-risk patients.^22^

Notably, the exploratory endpoint demonstrated poorer outcomes in those who had delayed implantation following CRT explant. It is possible that those with CRT devices explanted for an infective indication may have a greater burden of infective material owing to the presence of an LV lead, which may contribute to the poorer outcomes associated with delayed reimplantation. It may also be argued that an infective indication, whether this be systemic or local, may be an unidentified confounder. However, within each matched cohort there was not a survival difference depending on whether there was an infective indication for TLE and whether this was a systemic or local infection (Supplemental Figure 2). This suggests that the presence of infection was unlikely to be a confounder influencing this observation within the matched cohorts. Additionally, all patients had interrupted BiV pacing from time of TLE procedure to time of reimplantation. Most published work evaluates the acute implications of interrupting continuous BiV pacing. These studies have demonstrated that even brief interruptions in BiV pacing can result in worsening dyssynchrony and mitral regurgitation,^23^ left atrium and LV dimensions,^24^ and contractile reserve.^25^ Changes in cardiac biomarkers have also been associated with 48 hours of BiV interruption of CRT responders, with Rubaj and colleagues^26^ identifying a significant increase in proinflammatory cytokines and BNP concentrations. These findings may be a reason for the negative outcomes observed in this study associated with delayed reimplantation seen in the matched CRT cohort, but not observed in the matched non-CRT cohort.

### Limitations

Although the database collects many variables and allowed us to perform adjustments by PS matching, residual and unmeasured confounding within the matched and unmatched cohorts cannot be ruled out. Although our PS models were fitted based on several variables to foster adequate adjustments, we did not consider potential interactions among the covariates. The findings of our study are limited by the inherent issues identified with observational studies. Associations with mortality and hospitalization for the groups were discussed; however, the cause-and-effect relationship remains associative. Cause of death in these patients is unknown. We opted to only include patients who survived to discharge, which may have introduced survival and treatment bias. As our institution is a tertiary care center, referral bias could have affected the clinical data, thereby limiting generalization of these findings to other patient populations. The analysis on the impact of delayed reimplantation was performed within the matched cohorts, as the baseline characteristics of the CRT and non-CRT groups were similar after matching was performed. Within these constraints, a PS match analysis was considered an appropriate method of evaluating this hypothesis and potentially forms the basis of further investigation in the form of a randomized trial, which could more effectively reduce the potential number of unidentified confounders, which are often unavoidable as part of observational studies. As the baseline characteristics of the matched groups were very balanced, particularly with respect to the proportion of systemic and local infective indications for TLE, we believe there was justification for this comparison.

## Conclusion

The prognosis of patients with CRT who undergo TLE demonstrates similar mortality and hospitalization risk to non-CRT patients in a matched population. In an unmatched population, CRT patients had notably poorer outcomes and merit close follow-up post TLE procedures. There was increased risk of adverse outcomes associated with delayed reimplantation of CRT devices compared to other devices. This may be due to prolonged periods without continuous BiV pacing following TLE in patients with CRT devices, and this should be avoided where possible.

## Funding Sources

The work was supported by the Wellcome/EPSRC Centre for Medical Engineering [WT203148/Z/16/Z].

## Disclosures

J. Gould, M. Elliott, and V. Mehta have received fellowship funding from Abbott, outside of the submitted work. B. Sidhu is funded by NIHR and has received speaker fees from EBR Systems, outside of submitted work. J. Gould and M. Elliott have received project funding from Rosetrees Trust, outside of submitted work. S.A. Niederer receives research funding and/or consultation fees from Siemens, Abbott, and Pfizer outside of the submitted work. C.A. Rinaldi receives research funding and/or consultation fees from Abbott, Medtronic, Boston Scientific, and MicroPort, outside of the submitted work.

## Authorship

All authors attest they meet the current ICMJE criteria for authorship.

## Patient Consent

Patient consent for this study was not required owing to the use of de-identified and retrospective data.

## Ethics Statement

The database collection and analysis were approved by the Institutional Review Board of Guy’s and St Thomas’ Hospital. The research in this study was conducted in accordance with the Declaration of Helsinki.
