# Supplementary material for: Assessing long-term survival and hospitalization following transvenous lead extraction in patients with cardiac resynchronization therapy devices: A propensity score–matched analysis
Source: Heart Rhythm O2. 2021 Oct 30;2(6Part A):597–606. doi: 10.1016/j.hroo.2021.10.006 (PMC8703147; doi:10.1016/j.hroo.2021.10.006)
Supplement: Supplemental Table 1 [file mmc1.docx]

**Supplement**

**Supplementary table 1**

Baseline characteristics of the late and early reimplantation groups in the matched CRT and matched Non-CRT populations

| **Variable** | **Matched CRT Population** | | **Matched Non-CRT Population** | |
| --- | --- | --- | --- | --- |
|  | **Late Reimplantation** | **Early Reimplantation** | **Late Reimplantation** | **Early Reimplantation** |
| Total (n) | 78 | 114 | 74 | 118 |
| Follow-up time in months (median [IQR]) | 35.50 [18.50, 63.50] | 55.00 [26.00, 83.00] | 44.00 [23.75, 73.00] | 52.00 [29.25, 79.75] |
| Male (%) | 73 (93.6) | 87 (76.3) | 62 (83.8) | 98 (83.1) |
| Age at Explant (mean(SD)) | 69.17 (10.39) | 66.56 (11.61) | 70.04 (13.45) | 66.42 (12.43) |
| Time to reimplant (Days) | 13.00 [11.00, 18.00] | 0.00 [0.00, 0.00] | 13.00 [10.00, 19.75] | 0.00 [0.00, 3.75] |
| Reimplanted on same day | 0 (0.0) | 87 (76.3) | 0 (0.0) | 86 (72.9) |
| Reimplanted within 7 days | 0 (0.0) | 114 (100.0) | 0 (0.0) | 118 (100.0) |
| Lead Dwell Time (months) (median [IQR]) | 39.35 [14.00, 105.85] | 63.85 [34.20, 107.00] | 68.55 [24.60, 135.67] | 62.55 [29.47, 112.75] |
| Lead Dwell Time (Years) (median [IQR]) | 3.25 [1.20, 8.82] | 5.30 [2.85, 8.93] | 5.75 [2.05, 11.30] | 5.20 [2.50, 9.38] |
| CRTP (%) | 17 (21.8) | 36 (31.6) | - | - |
| CRTD (%) | 61 (78.2) | 78 (68.4) | - | - |
| ICD (%) | - | - | 37 (50.0) | 77 (65.3) |
| Pacing Device (%) | - | - | 7 (50.0) | 41 (34.7) |
| Local Infection (%) | 50 (64.1) | 25 (21.9) | 48 (64.9) | 33 (28.0) |
| Systemic Infection (%) | 21 (26.9) | 7 (6.1) | 20 (27.0) | 5 (4.2) |
| Any Infection (%) | 71 (91.0) | 32 (28.1) | 68 (91.9) | 38 (32.2) |
| Lead Dysfunction (%) | 7 (9.0) | 63 (55.3) | 3 (4.1) | 43 (36.4) |
| Functional Lead (%) | 0 (0.0) | 3 (2.6) | 0 (0.0) | 6 (5.1) |
| Lead Complication (%) | 1 (1.3) | 11 (9.6) | 1 (1.4) | 19 (16.1) |
| Lead Access (%) | 0 (0.0) | 8 (7.0) | 2 (2.7) | 20 (16.9) |
| Lead Pain (%) | 0 (0.0) | 2 (1.8) | 0 (0.0) | 2 (1.7) |
| Other indication (%) | 1 (1.3) | 10 (8.8) | 1 (1.4) | 22 (18.6) |
| No. of Previous Device Interventions (%) |  |  |  |  |
| 0 | 27 (34.6) | 39 (34.2) | 28 (37.8) | 34 (28.8) |
| 1 | 20 (25.6) | 29 (25.4) | 21 (28.4) | 46 (39.0) |
| 2 | 12 (15.4) | 25 (21.9) | 15 (20.3) | 19 (16.1) |
| 3 or more | 19 (24.3) | 21 (18.4) | 9 (13.6) | 19 (16.0) |
| LVEF (mean (SD)) | 38.41 (13.68) | 37.21 (11.51) | 40.12 (11.92) | 36.82 (14.11) |
| Ischaemic Heart Disease (%) | 49 (62.8) | 58 (50.9) | 41 (55.4) | 59 (50.0) |
| CABG (%) | 17 (21.8) | 20 (17.5) | 14 (18.9) | 23 (19.5) |
| Valve Disease (%) | 12 (15.4) | 11 (9.6) | 8 (10.8) | 17 (14.4) |
| Heart Failure (%) | 59 (75.6) | 81 (71.1) | 50 (67.6) | 91 (77.1) |
| Diabetes Mellitus (%) | 27 (34.6) | 15 (13.2) | 16 (21.6) | 22 (18.6) |
| Hypertension (%) | 47 (60.3) | 41 (36.0) | 33 (44.6) | 54 (45.8) |
| Peripheral Vascular Disease (%) | 5 (6.4) | 3 (2.6) | 3 (4.1) | 8 (6.8) |
| Stroke (%) | 8 (10.3) | 11 (9.6) | 8 (10.8) | 9 (7.6) |
| Chronic Respiratory Disease (%) | 12 (15.4) | 17 (14.9) | 7 (9.5) | 20 (16.9) |
| Chronic Kidney Disease (%) | 24 (30.8) | 29 (25.4) | 20 (27.0) | 29 (24.6) |
| Total Number of co-morbidities (%) |  |  |  |  |
| 0 | 3 (3.8) | 7 (6.1) | 5 (6.8) | 6 (5.1) |
| 1 | 9 (11.5) | 22 (19.3) | 14 (18.9) | 21 (17.8) |
| 2 | 15 (19.2) | 34 (29.8) | 17 (23.0) | 26 (22.0) |
| 3 | 16 (20.5) | 22 (19.3) | 15 (20.3) | 28 (23.7) |
| 4 | 12 (15.4) | 19 (16.7) | 14 (18.9) | 20 (16.9) |
| 5 | 13 (16.7) | 6 (5.3) | 4 (5.4) | 9 (7.6) |
| 6 | 10 (12.8) | 4 (3.5) | 4 (5.4) | 6 (5.1) |
| 7 | - | - | 1 (1.4) | 2 (1.7) |
| Creatinine Level [mg/dL] (mean (SD)) | 107.50 [86.00, 132.75] | 98.00 [80.00, 122.75] | 101.50 [85.00, 130.75] | 99.00 [81.25, 127.00] |
| eGFR [ml/min/1.73m2] (mean (SD)) | 61.56 (19.51) | 64.17 (20.30) | 61.89 (19.98) | 65.13 (20.25) |
| eGFR<60 ml/min/1.73m2 (%) | 35 (44.9) | 45 (39.5) | 32 (43.2) | 41 (34.7) |
| History of Previous Extraction (%) | 14 (17.9) | 13 (11.4) | 8 (10.8) | 17 (14.4) |
| Superior Approach (%) | 76 (97.4) | 113 (99.1) | 71 (95.9) | 118 (100.0) |
| Manual Traction Only (%) | 20 (25.6) | 16 (14.0) | 14 (18.9) | 16 (13.6) |
| Non powered tool only (%) | 17 (21.8) | 17 (14.9) | 12 (16.2) | 23 (19.5) |
| Powered tools only (%) | 5 (6.4) | 14 (12.3) | 4 (5.4) | 12 (10.2) |
| Inferior approach (%) | 13 (16.7) | 13 (11.4) | 13 (17.6) | 10 (8.5) |
| Complete Removal (%) | 70 (89.7) | 94 (82.5) | 67 (90.5) | 105 (89.0) |
| Partial Removal (%) | 4 (5.1) | 17 (14.9) | 6 (8.1) | 9 (7.6) |
| Clinical Failure (%) | 0 (0.0) | 2 (1.8) | 0 (0.0) | 1 (0.8) |
| Minor Complications (%) | 6 (7.7) | 9 (7.9) | 4 (5.4) | 11 (9.3) |
| Major Complications (%) | 1 (1.3) | 1 (0.9) | 2 (2.7) | 0 (0.0) |

*Abbreviations: CRTP: Cardiac Resynchronisation Therapy Pacemaker; CRTD: Cardiac Resynchronisation Therapy Defibrillator; ICD: Implantable Cardiac Defibrillator; CABG: Coronary Artery Bypass Graft; eGFR: estimated Glomerular Filtration Rate; LVEF: Left Ventricular Ejection Fraction*
