# Supplementary material for: Assessing long-term survival and hospitalization following transvenous lead extraction in patients with cardiac resynchronization therapy devices: A propensity score–matched analysis
Source: Heart Rhythm O2. 2021 Oct 30;2(6Part A):597–606. doi: 10.1016/j.hroo.2021.10.006 (PMC8703147; doi:10.1016/j.hroo.2021.10.006)

**Supplementary Figures**

**Figure 1 –** Histograms showing the density of propensity score distribution in the treated and control groups before and after matching.


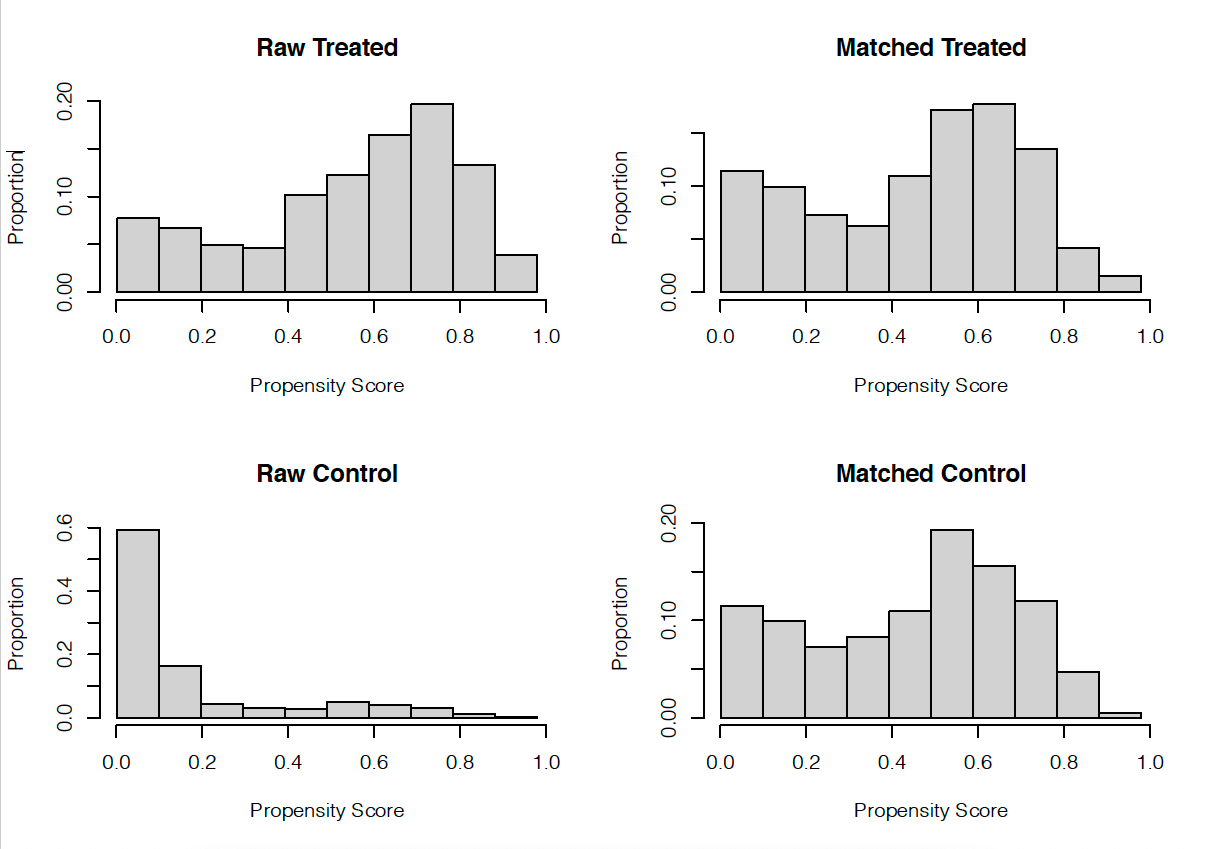


**Figure 2 –** Kaplan-Meier survival probability for mortality in patients depending on indication for TLE.

Figure 2A – Matched CRT group (Infective vs Non-Infective Indication)


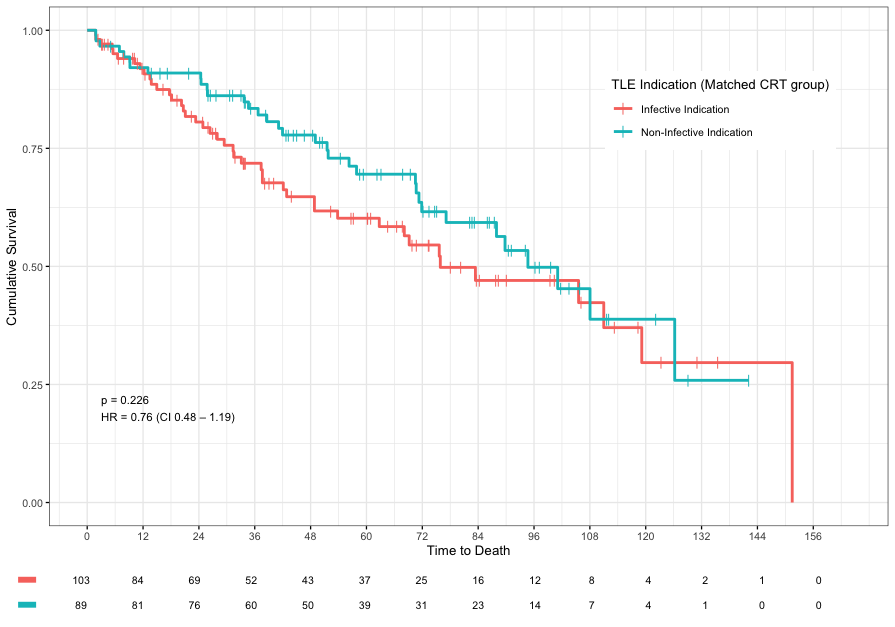


Figure 2B – Matched non-CRT group (Infective vs Non-Infective Indication)


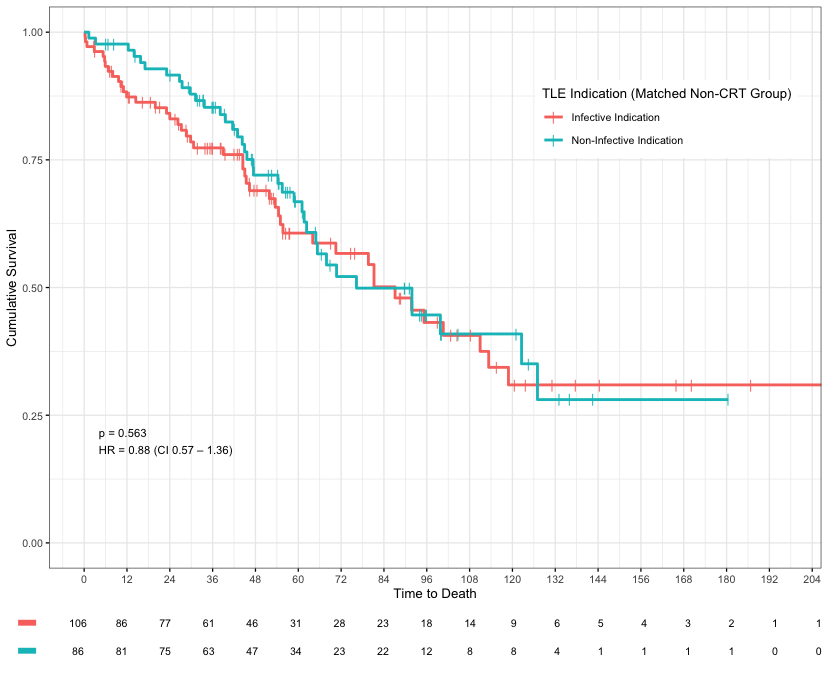


Figure 2C – Matched CRT Group (Systemic vs Pocket Infection)


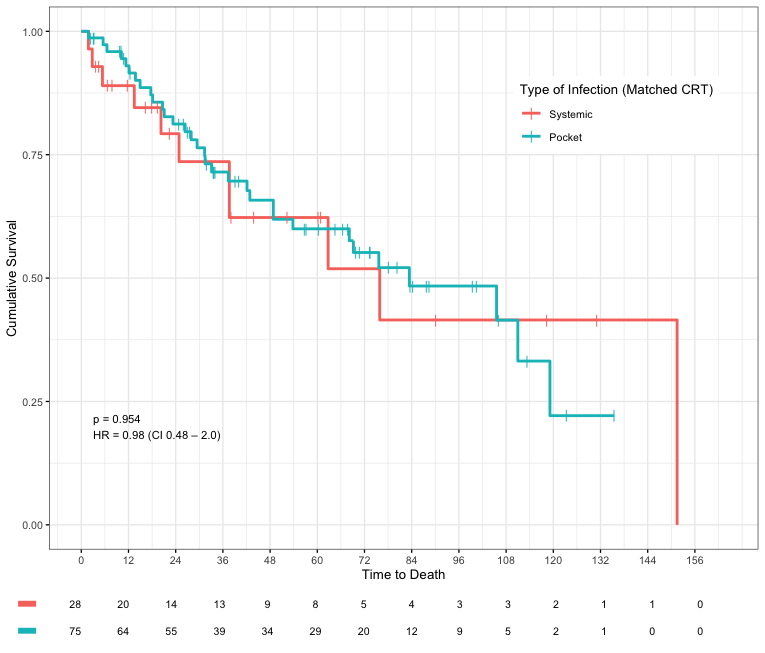


Figure 2D – Matched non-CRT group (Systemic vs Pocket Infection)


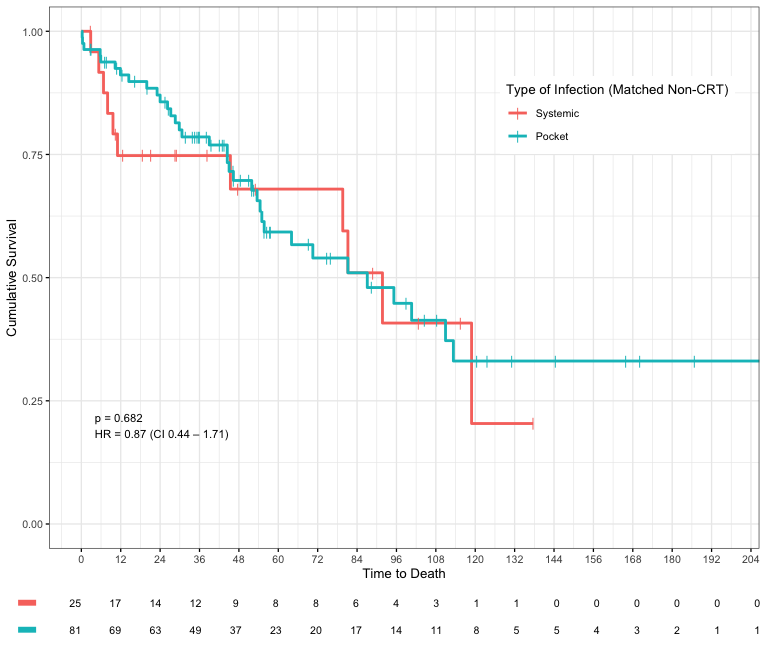

Supplement: Supplemental Figures [file mmc2.docx]
